# Innovative Synchronous Use of a Reverse Sural Flap and Reverse Hemisoleus Muscle Flap for Post-traumatic Lower Leg Reconstruction: A Case Report With a Long Term Follow-Up and a Quality of Life Assessment

**DOI:** 10.7759/cureus.69469

**Published:** 2024-09-15

**Authors:** Daniel J Rams, Maria Klimeczek-Chrapusta, Kacper Stolarz, Piotr Panek, Anna Chrapusta

**Affiliations:** 1 Malopolska Burns and Plastic Surgery Center, Ludwik Rydygier Memorial Hospital in Kraków, Kraków, POL; 2 Medicine, Jagiellonian University Medical College, Kraków, POL; 3 Malopolska Burn and Plastic Surgery Center, Ludwik Rydygier Memorial Hospital in Kraków, Kraków, POL

**Keywords:** surgery, flap technique, reconstruction, wound closure, limb salvage

## Abstract

We present a case report of a 47-year-old male with an extensive tissue deficiency of the right lower leg. The patient was hospitalized for approximately one month in the intensive care unit following a motorcycle accident that resulted in polytrauma. He suffered a fracture of frontal and parietal bones, traumatic brain injury, intracerebral hematoma with a subarachnoid hemorrhage and thoracic trauma. At first, lower leg wound was treated with a negative pressure wound therapy vacuum-assisted closure (VAC) dressing. Afterwards, he was qualified for a surgical wound closure with synchronous use of two reverse flow flaps: a reverse sural flap (RSF) and a reverse hemisoleus muscle flap (RHMF). Both flaps were dissected, and the RHMF was used to cover the exposed bone and the fracture site while the RSF closed the distal part of the wound. Split-thickness skin graft was meshed in scale of 1:1.5 and used to cover the RHMF and the remaining lower leg wounds. In the following days, uneventful wound healing was observed and the patient was discharged on day 34. The patient was invited for a follow-up examination two years after the procedure. His quality of life was assessed using SF-36 and Lower Extremity Functional Scale. It was determined to be satisfactory when compared to patients with identical injuries. Ultrasound examination of the gradient and blood flow velocity showed preserved graft perfusion and no structural abnormalities were detected. Adequate wound preparation and the choice of surgical technique allowed rapid healing and, above all, salvage of the limb that was at high risk of amputation.

## Introduction

Lower leg injuries with substantial muscle and skin loss, especially of the distal third, are difficult to manage due to the lack of soft tissue in this area and deficient vascularization. Despite recent advancements in microsurgery techniques, which have caused microsurgical free flaps to become a preferred method for managing such injuries, as they enable single-stage reliable coverage, they are associated with several drawbacks [1]. Patients with extensive lower leg injuries are very often road accident survivors with polytrauma, which deems them not fit enough for lengthy reconstructions due to their clinical condition. Additionally, the use of microvascular free tissue transfer is often linked to extended operation time and hospitalization because of the necessity for constant monitoring of the free flap. Utilization of this technique many times leads to the sacrifice of one of the major vessels of the lower limb. Furthermore, many hospitals do not have the trained personnel and equipment needed for performing such surgeries [2, 3].

With that being said, the use of pedicle based and local muscle flaps, emerges as a quality alternative, with benefits such as faster dissection, quicker transfer and similarity to the local tissue. Authors argue, that medically compromised polytrauma patients might not be suitable for lengthy microsurgical free tissue transfers. Therefore, shorter in duration pedicle based or local flap reconstruction stands out as a reliable option. Moreover, local flaps in the lower leg closely resemble the surrounding healthy tissue, unlike free flaps harvested from other regions [4]. 

We present a case report of a polytraumatized patient where a synchronous use of reverse sural flap (RSF) and reverse hemisoleus muscle flap (RHMF) was applied to cover a lower extremity tissue defect resulting from a road accident. 

The aim of this case study is to provide insight into this rarely reported flap technique for extensive soft tissue deficiency in the lower leg and assess long term outcomes of this surgery. This paper also highlights the synchronous use of two reversed flow flaps for lower limb salvage. 

## Case presentation

**Figure 1:**
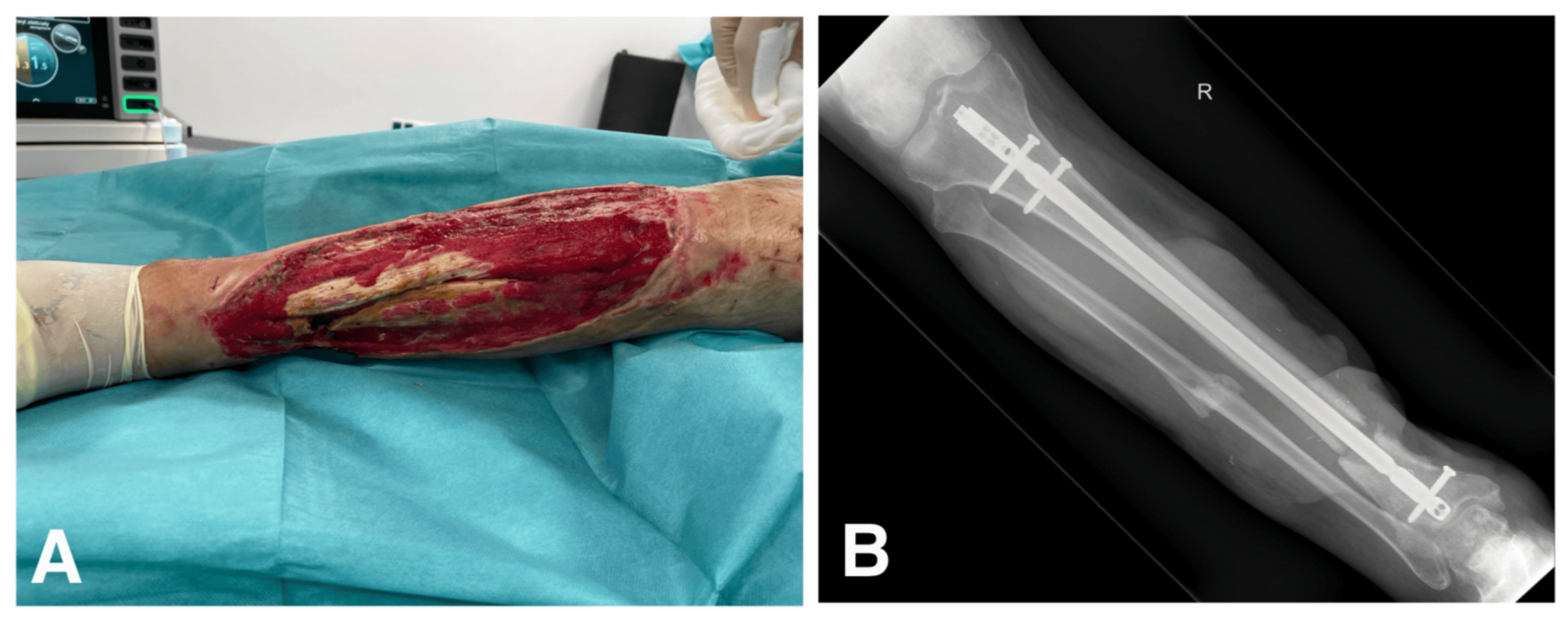
Extensive open wound of the lower leg with an apparent fracture (A), which was treated by immobilizing the tibial fracture with an intramedullary nail (B)

A 47-year-old male with an extensive tissue deficiency of the right lower leg had been hospitalized for around one month in an intensive care unit due to a polytrauma caused by a motorcycle accident. He suffered a fracture of frontal and parietal bones with a traumatic brain injury, intracerebral hematoma with a subarachnoid hemorrhage and thoracic trauma with ribs fracture. Current injuries to the right lower extremity showed an extensive open wound with an apparent fracture, which was treated by immobilizing the tibial fracture with an intramedullary nail by orthopaedic surgeons (Figure 1). Afterwards, he was handed over from the Neuroorthopaedics Department to a Burn and Plastic Surgery Department in June 2021. 

Upon admission, the patient was partially conscious. The lower leg wound was treated with a negative pressure wound therapy vacuum-assisted closure (VAC) dressing. A sample for bacteriological examination was collected. On the third day of hospitalization, he was qualified for surgical wound closure with synchronous use of two reverse flow flaps: RHMF and RSF. Afterwards, the patient was positioned in a lateral recumbent position, a meticulous debridement of the wound was performed. Next, both flaps were dissected with a reversed blood supply and rotated. 

**Figure 2:**
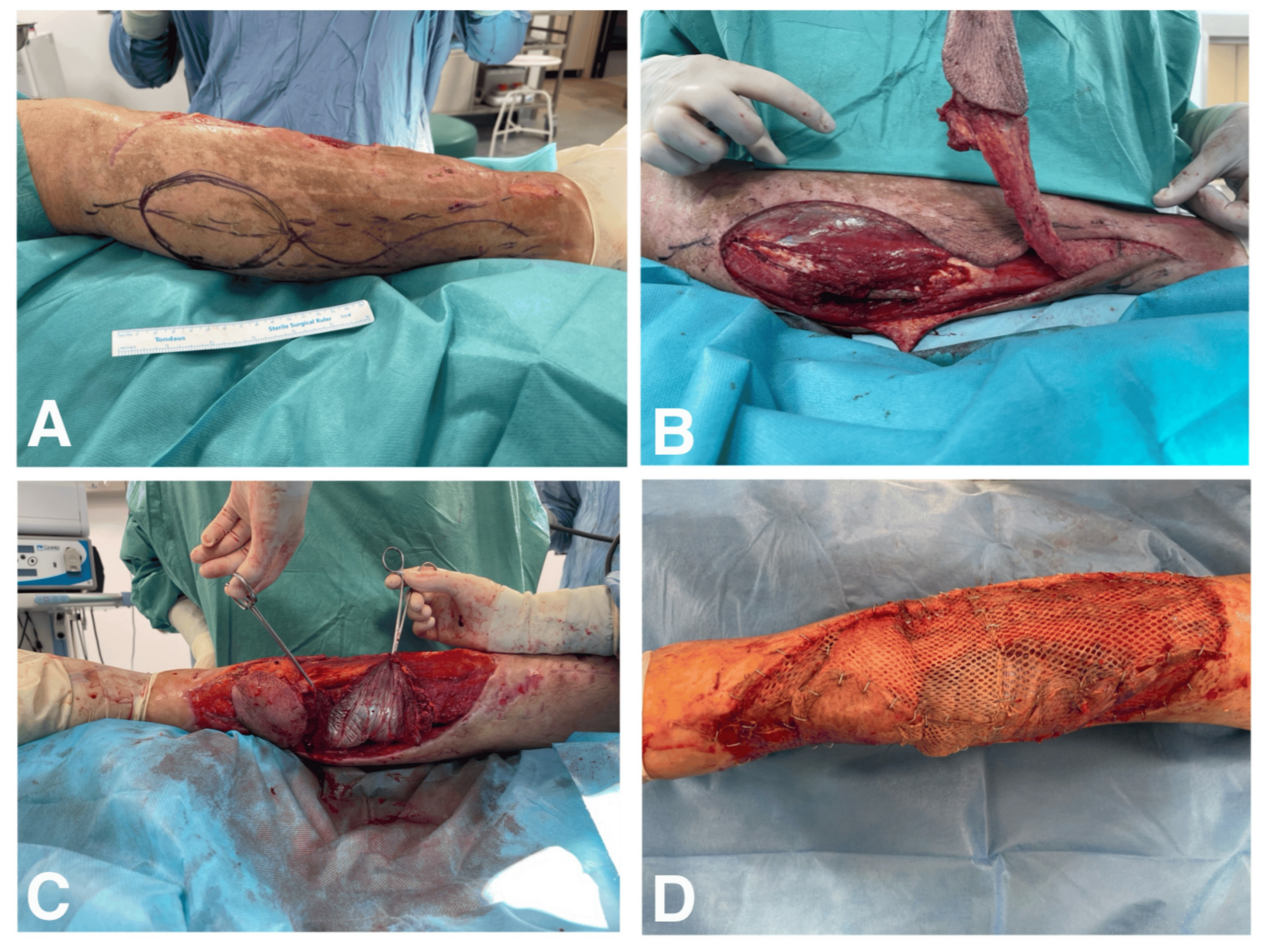
Individual stages of the procedure to close the vast tissue defect of the right lower leg with the RSF and the RHMF (A) Preparing for RSF grafting; (B) Dissected RSF; (C) Rotation of the RHMF with visible partially-completed defect reconstruction using RSF; (D) The remaining wounds were covered with intermediate-thickness skin grafts taken from the right thigh. RSF - reverse sural flap; RHMF - reverse hemisoleus muscle flap

The RHMF muscle flap was used to cover the exposed bones, including the fracture site, while the RSF fasciocutaneous flap closed the distal part of the wound. An intermediate split-thickness skin graft (STSG) was then harvested, meshed at a 1:1.5 ratio, and laid over both the muscle flap and the remaining wounds of the lower leg (Figure 2). On the following days, normal wound healing was observed. On postoperative day 14, during dressing changes, small areas of marginal necrosis in the RSMF region and isolated areas of lysis of STSG were observed. The devitalized tissues were removed, and the patient was qualified to have the residual wounds resealed with a STSG at a later date. Regular dressing changes on iodopovidone swabs were implemented.

**Figure 3:**
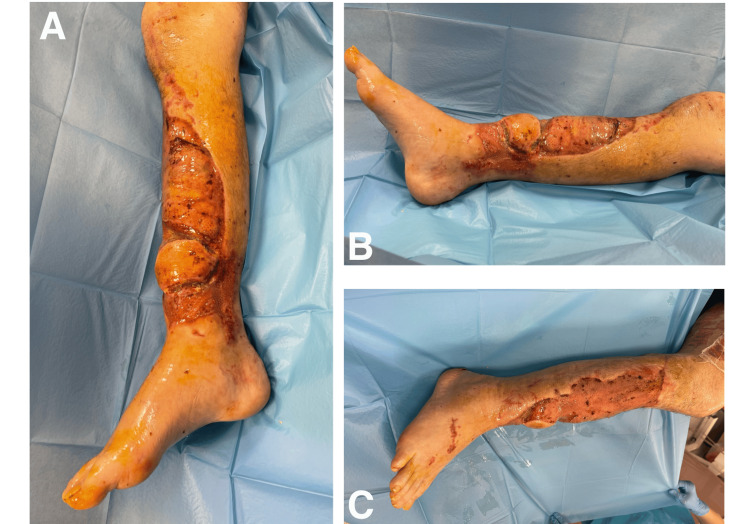
The condition of the wound healed the day before discharge (31 days after surgery) (A) View of the limb from the front; (B) View of the medial side of the limb; (C) View of the lateral side of the limb

From postoperative day 17, gradual verticalization of the patient was performed with intensive rehabilitation using a tall rollator walker. On the following days, dressings were changed daily, and on day 26, the residual wounds were closed with STSG assisted by negative pressure therapy. On day 34, the patient underwent follow-up laboratory and imaging examinations, where no significant abnormalities were found (Figure 3). The same day the patient was discharged home in good general and local condition.

**Figure 4:**
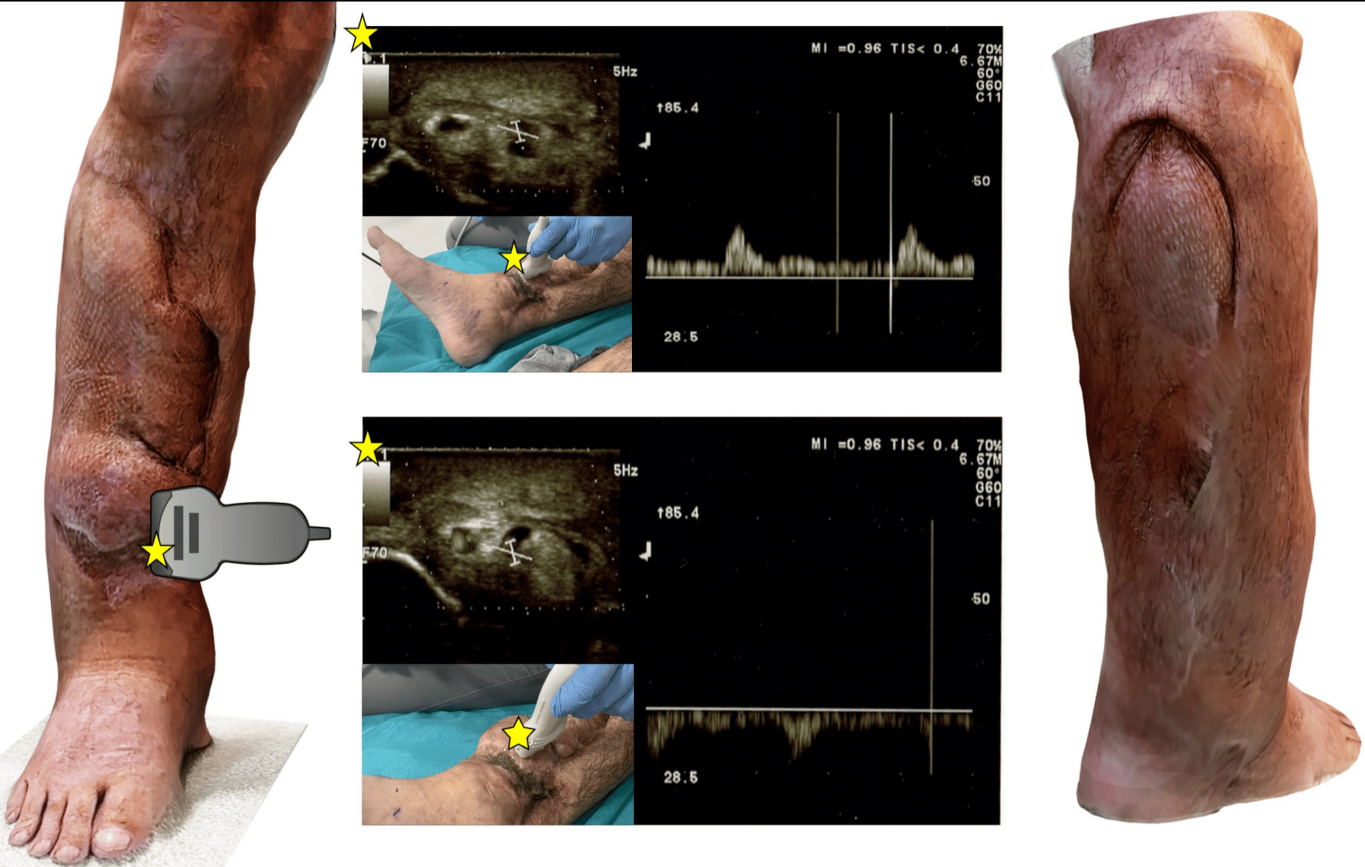
Summary of performed ultrasound imaging superimposed on a three-dimensional (3D) scan of the treated lower limb during a 33-month follow-up Yellow stars indicate the probe marker, and the arrowhead indicates the cephalic direction. Cross-section through the different levels on the anterior shin

**Figure 5:**
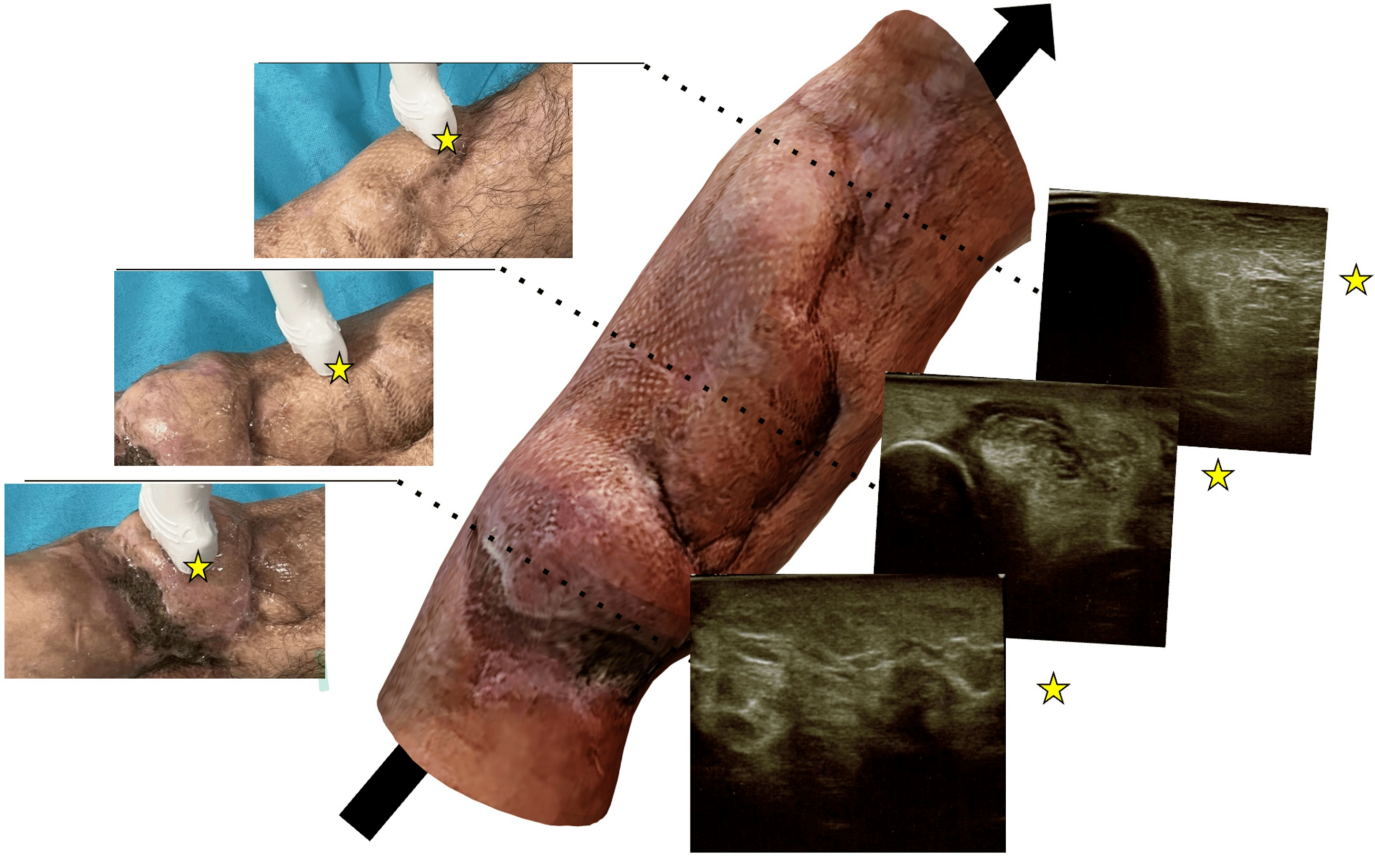
Summary of performed ultrasound imaging superimposed on a three-dimensional (3D) scan of the treated lower limb during a 33-month follow-up Doppler ultrasound in the flap pedicle. Visible retrograde flow spectrum

**Figure 6:**
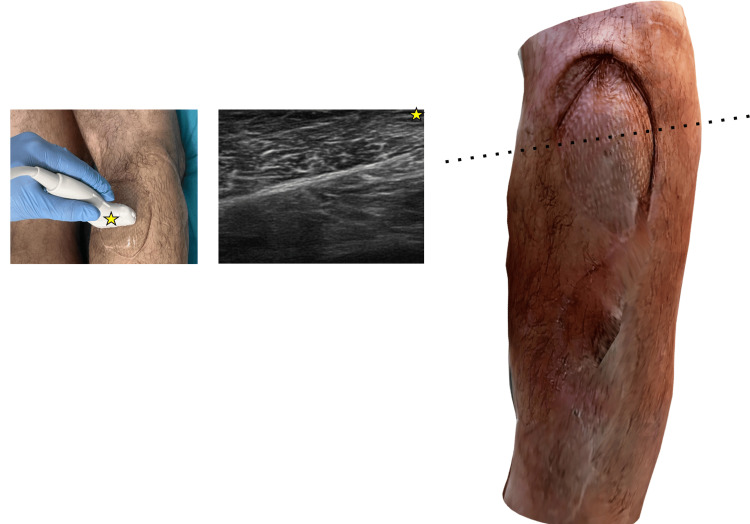
Summary of performed ultrasound imaging superimposed on a three-dimensional (3D) scan of the treated lower limb during a 33-month follow-up Cross-section through the donor site

A follow-up visit was conducted in March 2024, during which a series of physical and imaging examinations were performed, including a 3D scan of the lower limb (Figures 4- 6).

The patient indicates an improvement in his condition compared to his condition after discharge; having been rehabilitated many times. The patient was able to briefly put weight on the limb by walking several meters on his own, showed a compensatory gait, resembling a waddling gait and requiring support in the form of a walking cane. The Short-Form 36 (SF-36) and Lower Extremity Functional Scale (LEFS) questionnaire tests recorded scores of 40.0% (physical functioning) and 33.8%, respectively (Table 1).

**Table 1:** Summary of questionnaire studies performed during the 33-month follow-up

Name of the questionnaire survey	Percentage score
36-Item Short Form Health Survey (SF-36)	
Physical functioning	40.0%
Role limitations due to physical health	100.0%
Role limitations due to emotional problems	0.0%
Energy/fatigue	45.0%
Emotional well-being	40.0%
Social functioning	100.0%
Pain	67.5%
General health	25.0%
Health change	50.0%
Lower Extremity Functional Scale (LEFS)	
General score	33.8%

**Figure 7:**
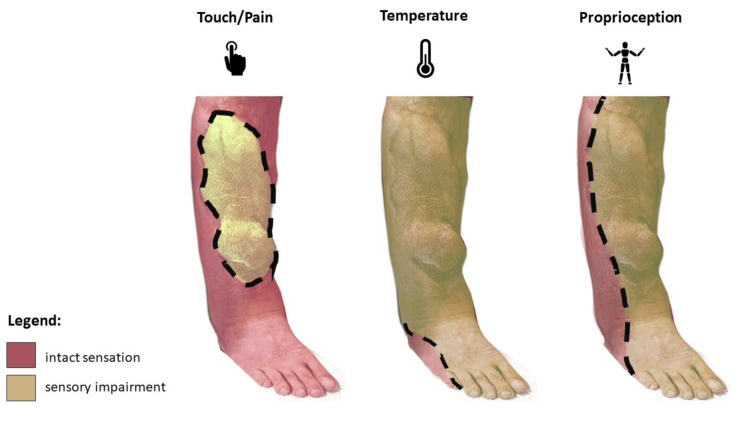
Summary of neurological assessment (touch and pain sensation, temperature sensing and propioreception) in a 33-month follow-up time-point yellow - areas of sensory impairment, red - areas with preserved sensation

The treated lower extremity was presented with no visible swelling, with a slightly enlarged ankle joint outline accompanying the presence of a properly healed wound containing superficially keratinized masses in the medial part of its lower margin. Physical examination revealed no complaints proximal to the lower leg, while the foot and ankle joint showed a positive Silfverskiöld test with slightly limited range of plantar flexion and restricted multiplanar movements in terms of inability to supinate and partially pronate the foot. On neurological examination, the greatest decrease in muscle strength was seen in supination with concomitant pain during deepened passive motion (Medical Research Council (MRC): 1/5), while the least was seen in plantar flexion of the foot (MRC: 4/5). There was a complete abolition of superficial sensation in the region of the flap-plasty and donor area (Figure 7). Moreover, an impaired sense of position and vibration in the full range of anterior compartment leg muscles motion was present, while sensation was preserved for lateral and posterior groups. A pulse present on the dorsal foot artery and posterior tibial artery with vivid vascular peristalsis of the flap was confirmed both by palpation and Doppler ultrasonography (USG). Subsequently, three-phase blood flow was visualized in the popliteal artery and pedicle vessels of the flap, i.e., calf vessels, both at rest and in elevation of the limb by 45° in the supine position. In addition, no excessive masses, signs of inflammation, thrombotic materials, or any other vascular pathologies were observed in USG.

## Discussion

Usually caused by road accidents, polytraumatic injury is directly life-threatening and requires immediate medical intervention [5]. The sequence of the actions taken is crucial, life-saving measures are always performed first, followed by addressing other injuries. In the described case, the patient was initially hospitalized in the Neuroorthopaedics Department, where vital functions and the most severe injuries were stabilized and taken care of, and then transferred to the Plastic Surgery Department, where the lower leg defect was managed.

Distal lower extremity injuries have always posed a unique challenge for reconstructive surgeons because of the soft tissue layer in this area being very thin, with far fewer subcutaneous compartments compared to other regions of the body. Furthermore, tendons are located superficially, and there is a lack of musculature capable of providing a sufficiently vascularized wound bed for healing [6, 7].

Therefore, classical teaching favors free microvascular tissue transfer for extensive soft tissue defects in the middle and distal third of the lower leg. However, polytraumatic patients, with obstacles such as being frail, medically compromised, or having suboptimal physiological conditions, seem especially difficult to be qualified for a lengthy microsurgical procedure [5, 7]. Moreover, many non-tertiary reference-level hospitals lack the necessary equipment or trained microsurgical staff to perform this type of procedure.

Our study describes a rarely reported, challenging surgical technique of synchronous application of two flaps in a polytraumatic patient in a life-threatening condition who was disqualified from a microsurgical free tissue transfer after a thorough medical assessment. After an in-depth literature search, two studies reporting similar techniques - a case report of synchronous use of medial hemisoleus flap and RSF [5] and an original study on the combined use of soleus and RSF [8] - were found. However, our study differs in terms of blood flow to the flaps. This is the first case report of simultaneous use of the RSF and RHMF for lower limb salvage. Therefore, we have presented a new technique that can be applied for lower leg reconstructions. 

RSF is a fasciocutaneous axial flap found to be reliable and versatile for the coverage of distal third wounds. Its arterial blood supply relies on the retrograde flow coming from the septocutaneous branches from the fibular artery and posterior tibial artery [4]. 

The soleus muscle, being the only type II muscle according to the Mathes and Nahai [9, 10, 11] classification in the distal part of the lower limb, allows the distal vascular pedicles to be cut off and the muscle to be rotated with the proximal dominant pedicle intact, which supports the choice of the RHMF.

RHMF, first described by Tobin et al. [11], is based on sural arteries arising from the popliteal artery and branches of the posterior tibial artery - distal perforators of the soleus muscle. Since it is a bipenniform flap, the lateral part of the muscle remains in situ, which reduces the amount of plantar flexion strength loss compared with a full soleus flap [12]. Moreover, the medial flap has a greater rotation angle compared to the conventional soleus muscle flap [1]. Due to the fact that the primary indication for RHMF is coverage of wounds of the distal medial leg smaller than 50 cm^2^ [13], these two flaps had to be combined.

A study by Thornton et al. [9, 14] reported that the use of a local muscle flap for reconstruction of a distal tibial wound appears to be more cost-effective than free-tissue transfer because of satisfactory outcomes at approximately half of the cost. An article by Pinsolle et al. [10] showed a shorter mean length of stay for patients with regional flaps used to cover wounds for coverage of the middle and distal third of the lower leg compared to those treated with free flaps. Additionally, their 15-year-long study reported that pedicled flaps were related to a lower rate and less severe complications than free flaps (18% vs 27%, respectively). Although the study by Pinsolle et al. [10] concluded that over the years of their study, their practice has changed to establishing pedicled flaps as a first choice to cover soft-tissue defects of the lower limb, they advocated for the use of free flaps for wide or composite defects.

Our study reports the long-term assessment of the results of this combined surgical technique. At the 33-month follow-up, a satisfactory condition of the healed wounds was observed, although the presence of keratinized masses suggests the existence of chronic irritation and potential deficiencies in regional hygiene. Trophic changes induced by ischemia were excluded since the absence of ulceration, the maintenance of body temperature, and the presence of USG confirmed viable peristalsis of vessels plays the role of an important indicator of proper circulation in the operated area. The sensory abnormalities observed in the operated area correlate to the distribution of dermatomes innervated by the corresponding spinal nerves. With regard to the substantial tissue interference used for reconstruction, such occurrences are considered probable surgical sequelae. Unfortunately, despite intensified treatment, the patient continues to experience some difficulties related to motor function. The predominance of plantar flexion over dorsiflexion of the foot is observed, as well as the inability to supinate the foot, which makes it difficult to maintain stable locomotor function. It is noteworthy that the performed Silfverskiöld test, indicating restriction of dorsiflexion of the foot with the knee straight and facilitation of flexion with the knee flexed, is positive secondary to the reconstructive surgery and the soleus muscle repositioning. One cannot ignore the fact that the patient suffered an extensive fracture of the bones of the right lower leg, the complications of which are an additional component of the observed walking difficulties, especially restrictions within the ankle joint [15, 16]. 

The issue of psychophysical health in patients after multi-organ trauma falls into a particularly difficult category, where, as mentioned, the main priority is to maintain vital functions due to the high mortality rate. Confirmation of this difficult situation can be found in the questionnaire studies conducted, which showed the presence of limitations in this sphere of the patient's health. The literature describes cases of treating injuries or defects in the distal lower limb with similar flap-plasty, yielding LEFS scores of about 45% [12] or 53% [13]. In a 2019 study by Schmidt et al. (2019) evaluating a shorter form of the SF-12 questionnaire, the percentage was 37% [14, 15, 16]. However, when comparing our patient's results (about 34% and 40%), which are lower than those in the literature, it should be taken into account that this holistic assessment also includes the consequences of extensive trauma and it seems impossible to assess the direct impact of the performed reconstruction on the patient's quality of life. A study conducted by Leland et al. [17] on 44 patients who underwent vascularized soft tissue reconstruction after lower extremity traumatic injuries reported 43.9 points for SF-36 scores in physical functioning and 46,4 in vitality, which is very similar to our 40 and 45 points in this categories respectively. Moreover, in comparison with that study [17], our patient scored higher in categories such as role limitations due to emotional well-being and social functioning, where he had absolutely no complaints. This proves that our technique of simultaneous RSF and RHMF application emerges as a good alternative to conventional methods used in these studies [14-17].

This case report highlights several key learning outcomes, namely: 1) the combined surgical approach in reconstructive surgery for lower extremity wounds in patients with polytrauma, 2) the surgical anatomy of the operated area with special attention to vascularization and innervation, 3) the course of long-term follow-up, 4) possible chronic complications and their impact on functional outcomes, and 5) the use of selected imaging techniques in assessing the patient's condition. In conclusion, the measures taken during treatment effectively prevented amputation of the injured limb, and the results obtained are within the range of results observed in the literature, taking into account the patient's history of polytrauma.

## Conclusions

This case report demonstrates a proposal that, to our knowledge, has not been reported before to close an open fracture of a lower leg with extensive soft tissue loss using a combined plastic surgery with two local flaps - a muscle flap (RHMF) and a fasciocutaneous flap (RSF). Adequate local wound preparation and choice of surgical technique allowed for rapid healing with the use of the flaps on retrograde blood inflow. Despite some limitations in the patient's psychophysical health, the more than 2.5-year postoperative follow-up showed satisfactory wound healing with preserved perfusion. The observed difficulties related to motor function, require continued rehabilitation, although the therapeutic measures taken effectively prevented the need for amputation of the injured limb, which is an added value for the patient.

Our approach not only allows medically compromised patients, who are unsuitable for lengthy procedures, to undergo limb-salvaging reconstruction after a large soft tissue defect, but also highlights a new pathway in treatment strategies for similar cases. By addressing the limitations, such as an inadequate size of a single local or pedicled flap, this method offers a solution for covering extensive defects, potentially providing a more viable option for patients who were previously considered inoperable.

Further research on a larger group of patients, who could undergo reconstruction with our proposed technique is needed to compare it with conventional flap techniques and validate its reliability for lower leg salvage. 
